# Impact of Cutting Data on Cutting Forces, Surface Roughness, and Chip Type in Order to Improve the Tool Operation Reliability in Sintered Cobalt Turning

**DOI:** 10.3390/ma17102210

**Published:** 2024-05-08

**Authors:** Emilia Franczyk, Wojciech Zębala

**Affiliations:** Department of Production Engineering, Faculty of Mechanical, Cracow University of Technology, 31-155 Cracow, Poland; wojciech.zebala@pk.edu.pl

**Keywords:** sintered cobalt, cutting forces, chips, surface roughness, tool geometry, tool operation reliability

## Abstract

The authors present the results of laboratory tests analysing the impact of selected cutting data and tool geometry on surface quality, chip type and cutting forces in the process of orthogonal turning of sintered cobalt. The selected cutting data are cutting speed and feed rate. During the experiments, the cutting speed was varied in the range of *v_c_* = 50–200 m/min and the feed rate in the range of *f* = 0.077–0.173 mm/rev. In order to measure and acquire cutting force values, a measuring setup was assembled. It consisted of a Kistler 2825A-02 piezoelectric dynamometer with a single-position tool holder, a Kistler 5070 signal amplifier and a PC with DynoWare software (Version 2825A, Kistler Group, Winterthur, Switzerland)). The measured surface quality parameters were *Ra* and *Rz*. The components of the cutting forces obtained in the experiment varied depending on the feed rate and cutting speed. The obtained test results will make it possible to determine the optimal parameters for machining and tool geometry in order to reduce the machine operating time and increase the life of the cutting insert during the turning of sintered cobalt, which will contribute to sustainable technology.

## 1. Introduction

In recent years, there has been a dynamic growth in technology across various industries, such as the aerospace and automotive industries, necessitating the development of better manufacturing techniques. These industries require high-quality products that are produced efficiently. Tool manufacturers implementing new solutions in terms of cutting tool geometry in order to improve their operation properties (increase the tool life), which in turn allows an improvement in cutting data and consequently a reduction in machine times necessary to complete the machining process [1], is one way of boosting the process efficiency. Monitoring physical phenomena such as cutting forces enables the detection of undesirable events. A sudden increase in cutting forces, while maintaining the same cutting data, is a signal of an anomaly for the operator, for instance, due to tool wear. Continuous monitoring reduces energy consumption, helps shorten production time and minimises downtime in the production process.

Cobalt or nickel alloys are often used in various industries, such as aerospace. These alloys are machined using conventional (e.g., turning) or nonconventional manufacturing techniques (e.g., EDM) [2]. Cobalt alloys have an outstanding resistance to corrosion and stress, making them suitable for aerospace, marine, medical and industrial applications [3,4]. They are recognised for their superior strength and hardness, which decrease slightly at higher temperatures. Usually, components are cast or sintered to low tolerances, and the final shape and finish are achieved by grinding [5,6]. Surface quality is of particular interest while machining such materials, as it affects the life and functionality. The machining is optimised in terms of surface roughness, inter alia *Ra* and *Rz* parameters, tool life and minimising the production costs and machine operating costs [7]. Due to their complexity, these alloys are difficult to machine, and coolant is often used during machining. Kshitij et al. described the need to eliminate economic problems resulting from cutting fluids in the machining industry [8].

Modifications in terms of tool geometry directly affect the forces occurring during the machining, which translates to the tool’s life. Franczyk et al. have shown that a drill geometry modification (bevelling) reduces the cutting forces by more than 20% [9]. Dogra et al. [10] have shown that the tool nose radius affects the roughness of machined surfaces and forces that arise during the cutting. In the paper presented by Neseli et al., the response surface methodology (RSM) was used, and a prediction model for surface roughness (average surface roughness *Ra*) was developed. The results indicated that tool geometry (the tool nose radius) was the dominant factor for the surface roughness [11].

Kopac et al. [12] found that the cutting speed parameter has the greatest impact on surface roughness, with higher cutting speeds resulting in better surface roughness values. The cutting forces increase as the feed rate increases, but not in a linear manner. Trung showed that increasing the cutting speed does not have as great an effect on cutting forces as increasing the feed rate [13]. Kluz et al. have shown that in dry turning, an increase in the feed rate leads to the appearance of a chemical reaction [14]. Grzesik et al. proved the influence of tool wear on friction resulting from thermal effects including thermal softening [15].

Research into controlling and shaping chips has been ongoing for many years, with ample literature available on the subject. However, the analysis and study of chips that form during the machining of hard-to-cut materials, used in various industries, are still vital areas of research. Salem et al. showed that chip morphology is influenced by cutting data: chip shape and type are directly influenced by the mechanical and physical properties of the machined material; chips become increasingly plastic as cutting speed increases; and higher cutting speeds produce more continuous chips [16]. Zhang et al. developed a finite element model of the turning process, which showed that cutting data and the type of tool geometry affect the type of chips produced. The authors then experimentally investigated and compared these two methods [17].

Efficiency is an important factor in machining, and one of the factors affecting the efficiency is tool life. Cutting tool wear is a process that starts at the first minute of tool operation in the material, because the tool operates at high mechanical and thermal loads. The tool loses its initial geometry, which affects the machining efficiency. Hoghoughi et al. observed that during turning, the most common wear phenomena are flank wear, abrasion, adhesion and the appearance of chipping [18]. In order to reduce the tool wear, the cutting data and tool geometry are optimised and appropriate coatings and lubricants are used in line with the tool manufacturer’s recommendations [19]. Grzesik et al. have compared the tool wear resistance with the use of the same coating but with a different stoichiometric ratio during the machining of a hard-to-cut material [20]. A reduction in operating wear means lower costs. N. Hirohisa has developed a cost estimation method, ensuring the minimum machining cost [21]. Anderberg et al. have proved that significant savings can be achieved if the material removal efficiency is increased thanks to optimised machining parameters [22]. Hui et al. have developed a model accounting for the stochastic nature of tool wear and activities related to tool maintenance, e.g., sharpening or tool replacement during the turning [23].

Based on the literature review, we developed a research plan to investigate the effect of the type of tool geometry and cutting data (*v_c_* and *f*) on roughness parameters *Ra*, *Rz*, cutting force and chip type during the turning of sintered cobalt. The authors have attempted to select the appropriate tool geometry and cutting data in order to significantly reduce the surface roughness, which will limit additional operations and improve the tool operation reliability, thus shortening the machine tool operation time.

## 2. Experiment Design

This section describes the workpiece material, the turning tools, and the test and measurement stands.

### 2.1. Workpiece Material

**Figure 1:**
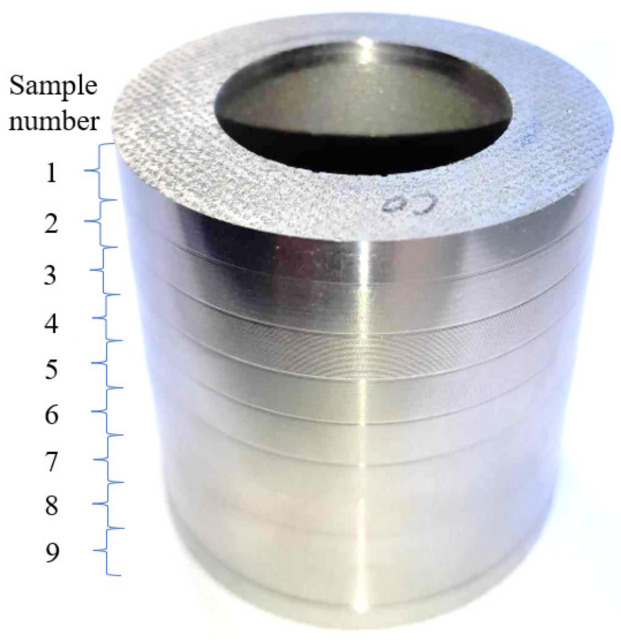
Research material—sintered cobalt.

The material used in the experiment was sintered cobalt bushings with a diameter of 48 mm. The chemical composition of sintered cobalt is shown in Table 1 and its physical properties are given in Table 2. The research material is shown in Figure 1.

### 2.2. Turning Tools

**Figure 2:**
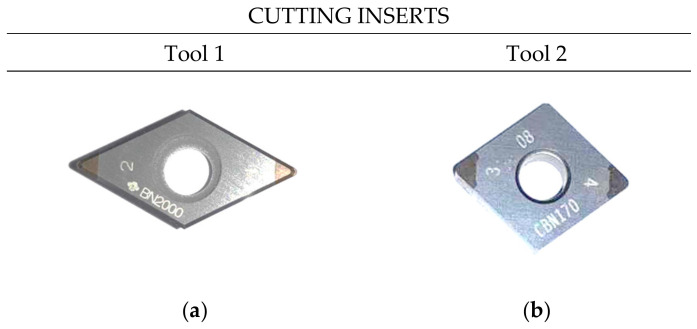
Tool 1 (**a**), tool 2 (**b**).

Two turning tools were used for the tests, which differed in the geometry of the cutting insert. Figure 2 shows the cutting inserts. The geometry of the cutting inserts is shown in Table 3. The tools have been assigned names: tool 1 and tool 2.

### 2.3. Test Stand, Measurement Methods

**Figure 3:**
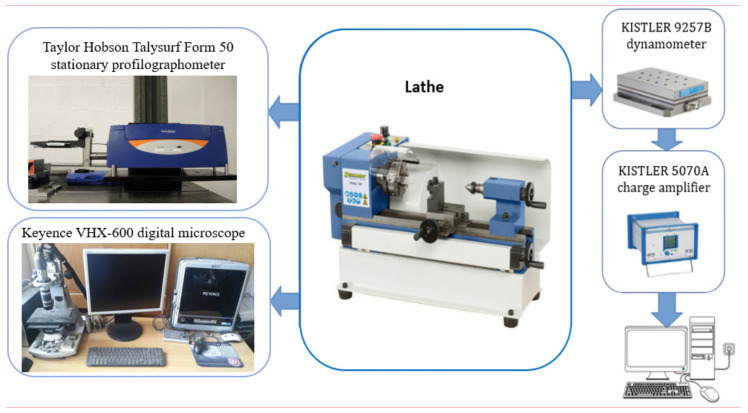
Test stand diagram.

The test stand consisted of four main elements: a Knuth Masterturn 400 lathe, a piezoelectric dynamometer, the Taylor Hobson Talysurf Form 50 stationary profilographometer, and a Keyence VHX-600 digital microscope. The scheme of the test stand is shown in Figure 3. The measurement of the cutting force values was carried out using a Kistler 9257B piezoelectric dynamometer connected to a PC through a Kistler 5070B charge amplifier (Kistler Group, Winterthur, Switzerland). The PC was equipped with DynoWare software (Version 2825A, Kistler Group, Winterthur, Switzerland).

### 2.4. Laboratory Tests

The test plan is shown in Table 4. The independent variables A and B were the cutting speed and the feed rate. The cutting speed and feed rate were at 3 levels, while the depth of cut (*a_p_*) was 0.2 mm. The tests included the turning of sintered cobalt with various cutting data and with different cutting inserts.

## 3. Experiment Results

### 3.1. Surface Finish

#### 3.1.1. Surface Roughness Measurements

Table 5 shows the 3D views of the examples of surfaces obtained as a result of using various tools.

#### 3.1.2. Microscopic Analysis of the Surface

Table 6 shows representative photos of surfaces obtained with the use of various tools. Examples of chipping are visible in the photos, marked in a circle with an arrow.

The microstructure analysis of the used turning tool leads to the conclusion that the studied parameters *Ra* and *Rz* are affected not only by the tool geometry but also by the cutting data.

In the case of machining with tool 1, the feed rate has no influence on *Ra* and *Rz*, while the cutting speed has a significant effect here. In the case of tool 2, the cutting speed has no influence on *Ra* and *Rz*, while the feed rate has a significant effect. Tool 1 has a smaller insert-included angle, and as a result, the profile peaks reflecting the tool shape overlap on the workpiece surface, giving a smooth surface, and consequently, the feed rate effect is reduced. In this case, the clearance angle is larger, deteriorating the heat removal and consequently reducing the tool life as the cutting speed increases. In the case of tool 2, which has a larger insert-included angle, the tool shape is better reflected in the material, and, as a result, the feed rate has a lesser effect on the surface roughness parameters *Ra* and *Rz*.

#### 3.1.3. Comparison of the Results 

**Figure 4:**
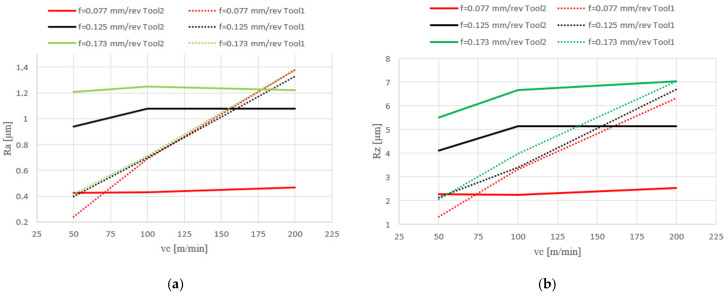
Surface roughness *Ra* (**a**) and *Rz* (**b**) vs. feed rate and cutting speed with various tools used.

Figure 4 shows the measured surface roughness parameters *Ra* and *Rz*, depending on the feed rate (*f*) and the cutting speed (*v_c_*) of various tools used.

#### 3.1.4. Statistical Analysis—Regression Equations

The results were analysed using the Minitab vol 17 software, which enables statistical analysis. The DOE option was selected in the program. The regression equation and analysis of variance were determined. Based on the equations, a surface plot was determined. The tested forces were *Fc*—cutting force; *Ff*—feed force; and *Fp*—radial force. The tested roughness parameters are *Ra*—the absolute average relative to the base length—and *Rz*, which measures the difference between the highest peak and lowest valley within the sampling length of five lines.

The equations for *Ra*, *Rz*, *Fc*, *Ff* and *Fp* regression for tool 1 are presented below:

*Ra =* 0.5592 − 10.93 *f* + 0.002061 *v_c_* + 83.02 *f·f* − 0.000008 *v_c_·v_c_* + 0.00539 *f·v_c_* (1)

*Rz =* 4.90 − 86.5 *f* + 0.00740 *v_c_* + 531.0 *f·f* − 0.000046 *v_c_·v_c_* + 0.0622 *f·v_c_* (2)

*Fc =* 8.3 + 486 *f* + 0.424 *v_c_* + 285 *f·f* − 0.001450 *v_c_·v_c_* + 0.132 *f·v_c_* (3)

*Ff =* 32.5 − 124 *f* + 0.256 *v_c_* + 1153 *f·f* − 0.001150 *v_c_·v_c_* + 0.851 *f·v_c_* (4)

*Fp =* 37.4 − 145 *f* + 1.483 *v_c_* + 2903 *f·f −* 0.00608 *v_c_·v_c_* + 1.157 *f·v_c_* (5)

The equations for *Ra*, *Rz*, *Fc*, *Ff* and *Fp* regression for tool 2 are presented below:

*Ra =* −1.521 + 30.05 *f* + 0.00281·*v_c_* − 86.2 *f·f −* 0.000008 *v_c_·v_c_ −* 0.00298 *f·v_c_* (6)

*Rz =* −3.94 + 79.7 *f* + 0.0171 *v_c_ −* 185.9 *f·f −* 0.000081 *v_c_·v_c_* + 0.0768 *f·v_c_* (7)

*Fc =* −49.9 + 1702 *f* + 0.212 *v_c_* − 4438 *f·f −* 0.000262 *v_c_.v_c_ −* 0.560 *f·v_c_* (8)

*Ff =* −93.1 + 2194 *f* + 0.335 *v_c_ −* 7562 *f·f −* 0.000567 *v_c_·v_c_* − 0.830 *f*·*v_c_* (9)

*Fp =* −203.8 + 4757 *f* + 0.713 *v_c_* − 15,563·*f* − 0.00143 *v_c_·v_c_* − 1.22 *f·v_c_* (10)

The results of the analyses are presented in Table 7 (for *Ra*), Table 8 (for *Rz*), Table 9 (for *Fc*), Table 10 (for *Ff*) and Table 11 (for *Fp*) for various tools. For each case, DF is degrees of freedom, Seq SS is sums of squares, Adj SS is the adjusted sums of squares, and Adj MS is the adjusted mean squares.

**Figure 5:**
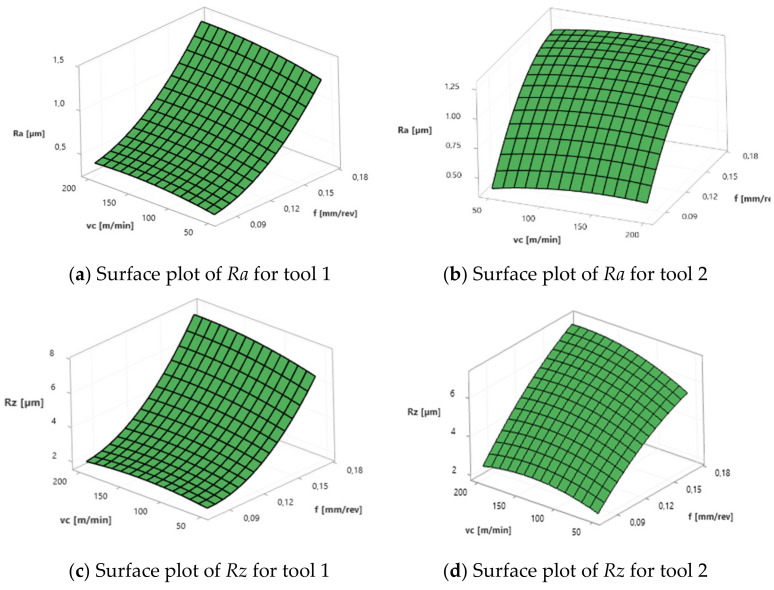

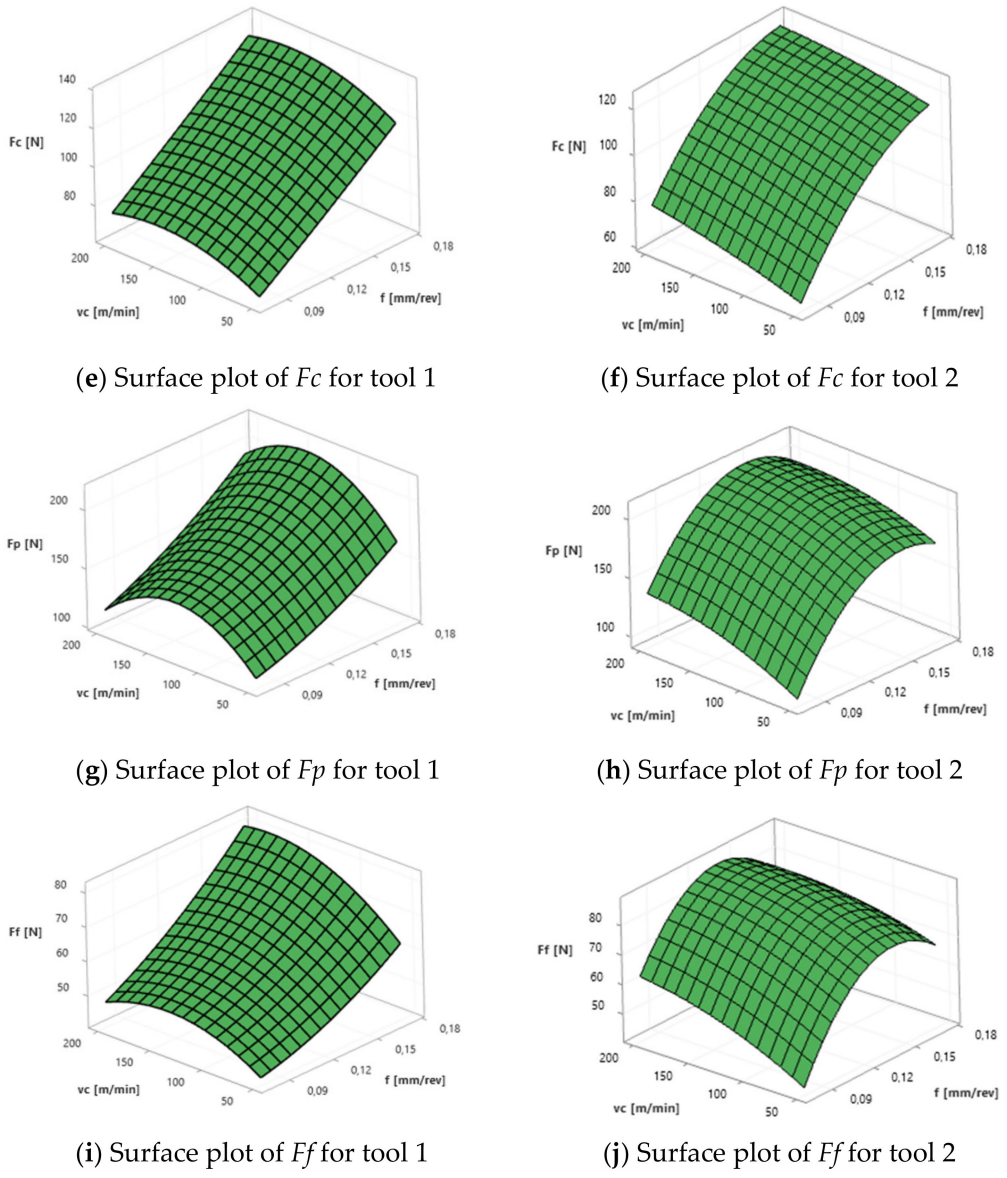
Surface and interval plots for *Ra*, *Rz*, *Fc*, *Ff*, *Fp* (**a**–**j**).

The plots produced from the regression equations are presented in Figure 5.

### 3.2. Chips

The next stage of research involved the classification of chips. Table 12 includes photographs of chips with classifications (“+” favourable, “0” acceptable, “-” unacceptable). A variety of chips are distinguished by different shapes and types. Chips are classified according to the Polish standard PN-IS. With tool 2, which has a smaller clearance angle, the chips are thinner and shorter, allowing for a better distribution of load along the cutting edge and more effective heat removal from the corner. This extends the tool life and allows better cutting data to be used, significantly increasing the productivity. Arc-type loose is the type of chip that forms when tool 2 is used and is classified as favourable (+), as it does not damage the tool or the workpiece.

In the case of tool 1, the chip is acceptable ”0” or unacceptable ”-“, because the washer-type helical chips are a hazard as they become tangled and damage the workpiece and the tool, necessitating additional machining operations in order to reduce the surface roughness parameters. In both cases, the increasing feed rate reduces chip breakage, which is beneficial because small chips spring back and as a result, they do not scratch the workpiece surface or damage the cutting insert.

## 4. Conclusions

The analyses were carried out to determine the impact of tool geometry, cutting speed and feed rate on the roughness values (*Ra*, *Rz*), cutting forces (*Fc*, *Fp*, *Ff*) and the type of chips in the longitudinal turning of sintered cobalt in order to shorten the time of machine operation.

The following conclusions can be made on the basis of the obtained results:

- The tool geometry affects the surface quality, which was proved by a microscopic analysis. Using a tool with a smaller included angle causes spalling on the workpiece surface, which generates costs because additional operations are needed, increasing the time of machine operation.
- As the feed rate increases, the forces *Fc* and *Fp* increase for both tools. For the smallest value of *v_c_* (50 m/min), the values of the cutting forces *Fc*, *Ff* and *Fp* are the smallest.
- As the feed rate increases, *Ra* and *Rz* increase for tool 2 but do not change significantly for tool 1, which is caused by the difference in the insert-included angle in these tools. The smaller the insert-included angle, the smaller the impact of the feed rate, because the profile peaks reflecting the tool shape overlap on the workpiece surface, giving a smooth surface.
- As the cutting speed increases, *Ra* and *Rz* values do not change significantly for tool 2 but increase for tool 1. The clearance angle is larger in this case, deteriorating the heat removal from the corner, so as the cutting speed increases, the tool life is reduced.
- The smallest roughness values occur at *v_c_* = 50 m/min and *f* = 0.077 mm/rev for both cutting tools, but this extends the duration of the process. At higher cutting speeds, other wear factors become more important, such as oxidation and diffusion resulting from an elevated temperature.
- Lower surface roughness (*Ra*, *Rz*) of the machined workpiece reduces the need for additional machining operations, thus shortening the time of machine operation.
- The most favourable chips are obtained at a higher feed rate and cutting speed, because chips are arc-type loose, which reduces the likelihood of damaging the surface of the workpiece and cutting insert.
- More favourable chips are produced when cutting with tool 2 because the chips are shorter in comparison with tool 1.
- A smaller clearance angle makes the chips thinner and shorter, allowing for a better distribution of load along the cutting edge. This extends the tool life and allows better cutting data to be used, significantly increasing the productivity.

After conducting research and analysing the available literature, it can be concluded that the studied problem is still valid, and its solution may contribute to the quality of manufacturing of items, improve the tool operation reliability and also reduce the time of machine operation in various industries.

## Figures and Tables

**Table 1:** Chemical composition of sintered cobalt.

	W	Mn	Si	P	Cr	Co	Mo	B	Ti	Fe	K
Result [%]	0.8	1.00	1.00	0.02	22.78	Balance	0.9	0.010	0.06	0.054	0.04

**Table 2:** Physical properties of sintered cobalt.

Particle Size Range	Morphology	Particle Size Distribution	Angle of Repose	Apparent Density	
53 μm	Spherical	33 μm	<40°	4.4 g/cm^3^	

**Table 3:** Cutting inserts’ geometry: tool 1 and tool 2.

	Tool 1	Tool 2
Insert included angle [^o^]	60	80
Corner radius [^o^]	0.8	0.8
Insert thickness [mm]	4.76	4.76

**Table 4:** Test plan.

Sample Number	Independent Variables		*f* (mm/rev)	*v_c_* (m/min)
	A	B		
1	1	1	0.077	50
2	1	2	0.077	100
3	1	3	0.077	200
4	2	1	0.125	50
5	2	2	0.125	100
6	2	3	0.125	200
7	3	1	0.173	50
8	3	2	0.173	100
9	3	3	0.173	200

**Table 5:** Three-dimensional views of the surface roughness examples obtained with various tools.

Specimen Number	3D Surface—Tool 1	3D Surface—Tool 2
1	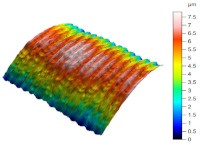	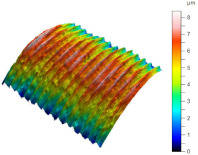
2	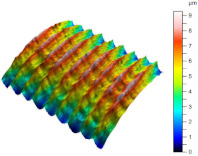	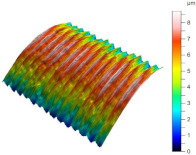
3	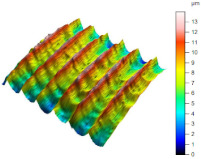	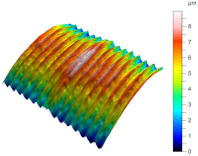
4	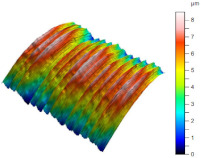	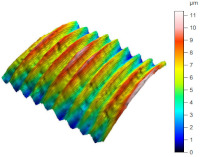
5	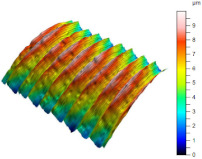	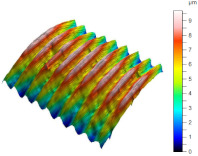
6	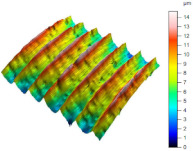	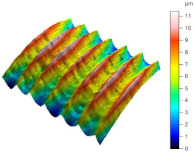
7	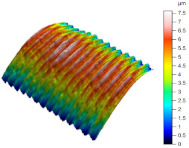	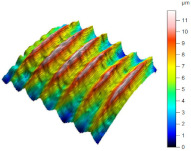
8	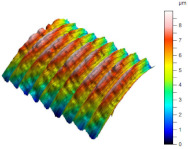	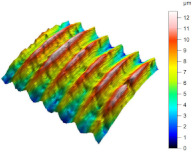
9	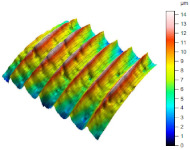	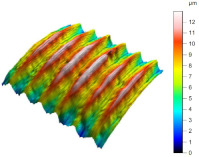

**Table 6:** Microscopic views of the surfaces obtained with various tools.

Parameters	Tool 1	Tool 2
*f* = 0.077 [mm/rev] *v_c_* = 50 [m/min]	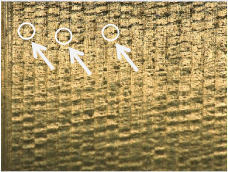	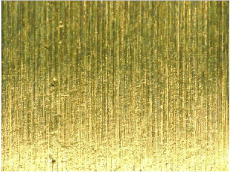
*f* = 0.077 [mm/rev] *v_c_* = 200 [m/min]	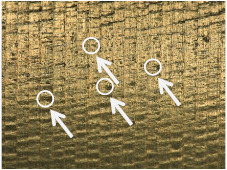	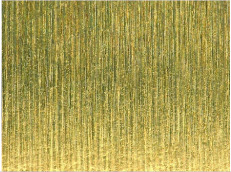
*f* = 0.125 [mm/rev] *v_c_* = 50 [m/min]	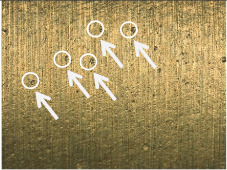	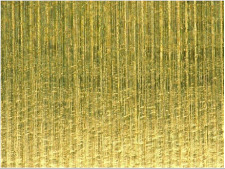
*f* = 0.125 [mm/rev] *v_c_* = 50 [m/min]	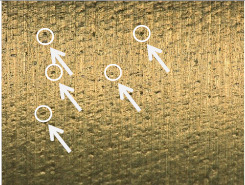	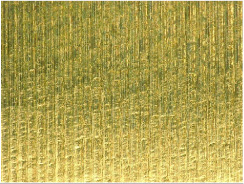
*f* = 0.125 [mm/rev] *v_c_* = 200 [m/min]	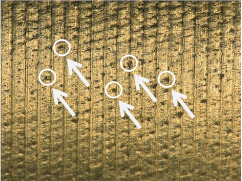	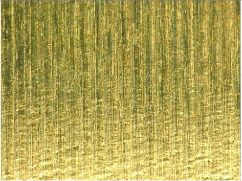
*f* = 0.173 [mm/rev] *v_c_* = 100 [m/min]	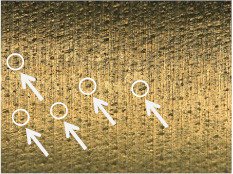	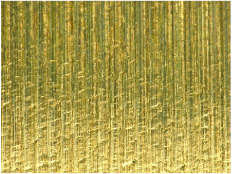

**Table 7:** ANOVA—*Ra.*

		Tool 1				Tool 2			
Source	DF	Adj SS	Adj MS	F-Value	*p*-Value	Adj SS	Adj MS	F-Value	*p*-Value
Regression	5	4.82575	0.965149	1236.83	0.000	1.00843	0.201686	91.47	0.002
*f*	1	0.05768	0.057675	73.91	0.000	0.14535	0.145346	65.92	0.004
*v_c_*	1	0.00754	0.007538	9.66	0.005	0.00468	0.004677	2.12	0.241
*f·f*	1	0.21952	0.219523	281.32	0.000	0.07880	0.078805	35.74	0.009
*v_c_·v_c_*	1	0.00857	0.008568	10.98	0.003	0.00312	0.003120	1.42	0.320
*f·v_c_*	1	0.00468	0.004680	6.00	0.023	0.00048	0.000476	0.22	0.674
Error	21	0.01639	0.000780			0.00661	0.002205		

**Table 8:** ANOVA—*Rz.*

		Tool 1				Tool 2			
Source	DF	Adj SS	Adj MS	F-Value	*p*-Value	Adj SS	Adj MS	F-Value	*p*-Value
Regression	5	129.581	25.9161	183.15	0.000	26.7647	5.35295	59.74	0.003
*f*	1	3.617	3.6167	25.56	0.000	1.0226	1.02265	11.41	0.043
*v_c_*	1	0.097	0.0971	0.69	0.417	0.1723	0.17232	1.92	0.260
*f·f*	1	8.979	8.9793	63.46	0.000	0.3669	0.36694	4.10	0.136
*v_c_·v_c_*	1	0.304	0.3041	2.15	0.157	0.3189	0.31894	3.56	0.156
*f·v_c_*	1	0.623	0.6230	4.40	0.048	0.3170	0.31697	3.54	0.157
Error	21	2.971	0.1415			0.2688	0.08960		

**Table 9:** ANOVA—*Fc.*

		Tool 1				Tool 2			
Source	DF	Adj SS	Adj MS	F-Value	*p*-Value	Adj SS	Adj MS	F-Value	*p*-Value
Regression	5	14,364.3	2872.87	54.08	0.000	12,808.0	2561.59	29.23	0.000
*f*	1	113.9	113.89	2.14	0.158	1399.4	1399.40	15.97	0.001
*v_c_*	1	318.5	318.51	6.00	0.023	79.5	79.48	0.91	0.352
*f·f*	1	2.6	2.58	0.05	0.828	627.3	627.30	7.16	0.014
*v_c_·v_c_*	1	304.3	304.27	5.73	0.026	9.9	9.90	0.11	0.740
*f·v_c_*	1	2.8	2.83	0.05	0.820	50.6	50.59	0.58	0.456
Error	21	1115.5	53.12			1840.4	87.64		

**Table 10:** ANOVA—*Ff.*

		Tool 1				Tool 2			
Source	DF	Adj SS	Adj MS	F-Value	*p*-Value	Adj SS	Adj MS	F-Value	*p*-Value
Regression	5	3701.15	740.231	15.76	0.000	12,808.0	2561.59	29.23	0.000
*f*	1	7.39	7.394	0.16	0.696	1399.4	1399.40	15.97	0.001
*v_c_*	1	116.49	116.491	2.48	0.130	79.5	79.48	0.91	0.352
*f·f*	1	42.31	42.312	0.90	0.353	627.3	627.30	7.16	0.014
*v_c_·v_c_*	1	191.41	191.413	4.08	0.056	9.9	9.90	0.11	0.740
*f·v_c_*	1	116.72	116.715	2.49	0.130	50.6	50.59	0.58	0.456
Error	21	986.07	46.956			1840.4	87.64		

**Table 11:** ANOVA—*Fp.*

		Tool 1				Tool 2			
Source	DF	Adj SS	Adj MS	F-Value	*p*-Value	Adj SS	Adj MS	F-Value	*p*-Value
Regression	5	27,489.7	5497.94	35.36	0.000	33,865.8	6773.2	22.82	0.000
*f*	1	10.2	10.19	0.07	0.800	10,927.9	10,927.9	36.82	0.000
*v_c_*	1	3902.4	3902.38	25.10	0.000	901.9	901.9	3.04	0.096
*f·f*	1	268.4	268.45	1.73	0.203	7714.2	7714.2	25.99	0.000
*v_c_·v_c_*	1	5352.1	5352.14	34.42	0.000	295.1	295.1	0.99	0.330
*f·v_c_*	1	215.8	215.80	1.39	0.252	241.4	241.4	0.81	0.377
Error	21	3265.2	155.48			6231.9	296.8		

**Table 12:** Chip classification for different *f* and *v_c_* values.

	*f* = 0.077 mm/rev *v_c_* = 200 m/min	*f* = 0.125 mm/rev *v_c_* = 200 m/min	*f* = 0.125 mm/rev *v_c_* = 100 m/min
**Tool 1**	Washer-type helical long (-)	Arc-type loose (0)	Washer-type helical long (-)
	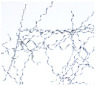	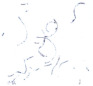	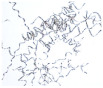
**Tool 2**	Arc-type loose (+)	Arc-type loose (+)	Arc-type loose (+)
	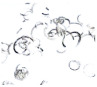	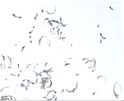	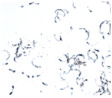

## Data Availability

The original contributions presented in the study are included in the article, further inquiries can be directed to the corresponding authors.
